# Cardiometabolic Effects of Ilex paraguariensis (Yerba Mate): A Narrative Review of Evidence and Research Gaps

**DOI:** 10.7759/cureus.114641

**Published:** 2026-08-17

**Authors:** Krystian Czernikiewicz, Piotr Frak, Urszula Kowalska-Frak, Agata Lemanska, Maja Karminska

**Affiliations:** 1 General Practice, F. Raszeja Municipal Hospital, Poznań, POL; 2 General Medicine, H. Cegielski-Poznań (HCP) Medical Centre, Poznań, POL; 3 Orthodontics and Dentofacial Orthopedics, University Center of Dentistry and Specialized Medicine, Poznań, POL; 4 General Medicine, Wojewódzki Szpital Wielospecjalistyczny im. dr. Jana Jonstona w Lesznie, Leszno, POL; 5 Internal Medicine, Prof. S. T. Dabrowski Hospital in Puszczykowo, Poznań, POL

**Keywords:** cardiometabolic, glucose metabolism, ilex paraguariensis, lipid profiles, mate tea, metabolic, obesity, yerba mate

## Abstract

Cardiovascular and metabolic diseases remain a major global health burden, yet their development is linked to modifiable risk factors. Available pharmacotherapy can effectively mitigate these risk factors but is associated with potential side effects. This motivates the search for complementary benefits offered by plant-derived substances. Yerba mate (YM) is a traditional South American beverage derived from crushed *Ilex paraguariensis* (IP) leaves. The infusion is rich in bioactive compounds such as polyphenols, flavonoids, and alkaloids known for their beneficial effects on metabolism. The aim of this review is to summarize current evidence on the cardiometabolic benefits of YM infusion and to outline current evidence gaps. The effects of YM on the human body appear to be multidirectional. The consumption of YM is associated with a reduction in postprandial glucose, as well as overall glycemic control and beneficial effects on endothelial function and a reduction in oxidative stress, along with an unclear effect on blood pressure. Additionally, though more modest, a beneficial influence on lipid profile has been found in preclinical studies, with more subtle changes recorded in humans. YM seems to have beneficial effects on postmenopausal metabolic status. The safety profile of YM is favorable, and available preclinical evidence suggests that yerba mate is generally well-tolerated and associated with a relatively low risk of adverse effects. These observations highlight the need for further adequately designed studies to achieve a more comprehensive understanding of the health effects of YM infusion.

## Introduction and background

Contemporary societies face a growing burden of metabolic and cardiovascular disorders, which have a multidimensional impact on human health, quality of life, and morbidity.

Based on World Health Organization (WHO) data, cardiovascular diseases are the leading cause of death globally, with 19.8 million deaths in 2022 alone, which represents 32% of all deaths worldwide. The risk of most of these diseases might be mitigated by the proper management of behavioral and environmental risk factors, including tobacco use, unhealthy diet (including excess salt, sugar, and fats), obesity, physical inactivity, the harmful use of alcohol, and air pollution [1].

According to the World Health Organization (WHO), 2.5 billion adults were overweight in 2022, of whom 890 million were living with obesity; 43% of the adult population was overweight and 16% obese, a prevalence that has more than doubled since 1990, while adolescent obesity has quadrupled. A higher-than-optimal body mass index (BMI) caused an estimated five million deaths in 2019 [2]. Obesity rarely occurs in isolation: it is comorbid with dyslipidemia, hyperglycemia, hypertension, and insulin resistance, together constituting a metabolic syndrome that includes type 2 diabetes mellitus (T2DM) and metabolic dysfunction-associated steatohepatitis (MASH) [3]. On the other hand, there is a parallel burden of diabetes. The proportion of adults living with diabetes climbed from 7% in 1990 to 9% in 2014, to 14% in 2022, surpassing 800 million people worldwide, and the disease was the direct cause of 1.6 million deaths in 2021 [4,5].

The global burden of the mentioned diseases continues to rise, creating an urgent demand for safe, accessible, and effective interventions, which might differ from widely known and accepted mechanisms of action. Available pharmacotherapies, although effective, are costly and carry notable gastrointestinal, cardiovascular, and psychiatric risks [3,6]. These limitations drive a growing interest in identifying novel, safe, and multitargeted substances, including those of plant origin. Rather than serving as alternatives to current standards of care, they are envisioned as novel adjuncts capable of expanding existing therapeutic strategies.

One such candidate is yerba mate (YM) (*Ilex paraguariensis *{IP}), a traditional South American infusion, which has emerged as a particularly promising, yet overlooked, candidate. It is prepared from the dried, ground leaves of the yerba mate holly, steeped in hot water in a gourd (calabash) and traditionally drunk through a metal straw with a built-in filter (the bombilla). Its leaves are rich in bioactive compounds such as polyphenols, including chlorogenic and caffeic acids, flavonoids such as rutin and quercetin, purine alkaloids (caffeine, theobromine, and theophylline), and triterpenoid saponins [7-11]. A growing body of research exhibits antioxidant and anti-inflammatory activity, influences thermogenesis and adipose tissue remodeling, and supports mitochondrial function [7,12,13]. In both preclinical models and human trials, it has been reported to reduce body weight, body-fat mass, and abdominal adiposity, without significant adverse effects [6,12]. It has been shown to improve glycemic control [14]. It has additionally been credited with hypocholesterolemic and cardioprotective effects [6,8]. Collectively, these observations have established YM as a genuinely metabolically active beverage with therapeutic potential in obesity and related disorders.

While several reviews have addressed individual aspects of YM’s biological activity, evidence on its cardiometabolic effects remains scattered across preclinical and clinical studies of variable quality. This review aims to synthesize current evidence on the cardiometabolic effects of YM infusion and to identify the main gaps limiting the translation of these findings into clinical recommendations.

## Review

Materials and methods

A thorough search of publicly available PubMed and SciELO databases was conducted to identify articles relevant to the review topic. The databases were searched in June 2026. Search strings used to extract articles consisted of keywords that were combined with the Boolean operators AND and OR: “yerba mate”, “*Ilex paraguariensis*”, “metabolism”, “cardiovascular”, “glucose”, “cardiovascular diseases”, “endothelial function”, “atherosclerosis”, “lipid metabolism”, “glycemic control”, and “diabetes mellitus” in both human and animal studies. The research had a specific timeframe from 2015 to 2025 in order to extract the latest data. Additional targeted searches were conducted to identify evidence regarding GLP-1 signaling, incretin biology, gut microbiota, and polyphenol bioavailability to explore areas with limited evidence. The aforementioned method was designed to maximize the number of results and provide a comprehensive literature search. To optimize the search outcomes, the search terms were modified by adding modifiers that allow searching using MeSH terms, as well as searching directly in titles and abstracts.

**Figure 1:**
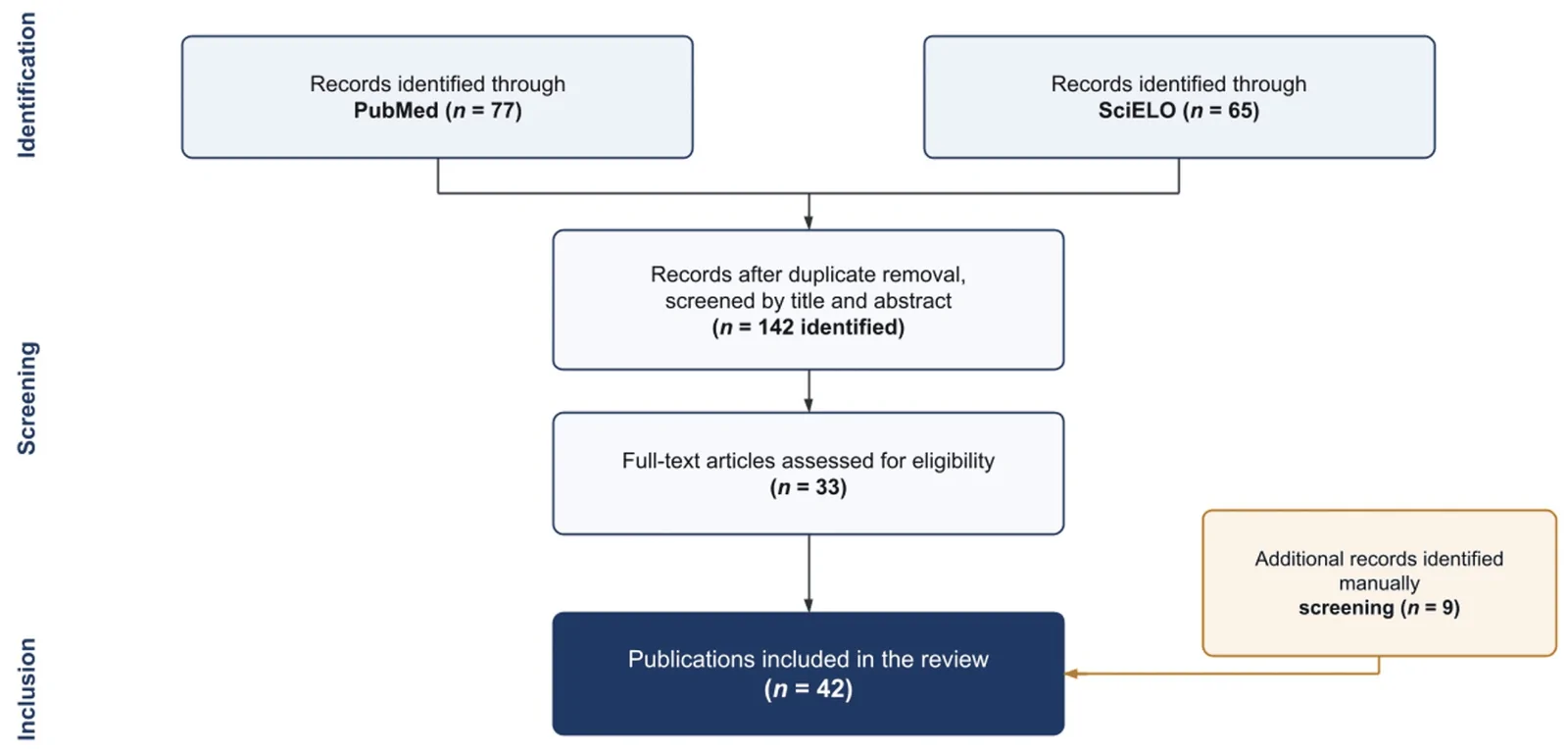
Study-selection flow diagram.

The inclusion criteria included all types of articles (though clinical trial articles and meta-analyses were prioritized), human and nonhuman models in clinical trials, and reviews. Article language was restricted to English only; however, the SciELO database was searched to incorporate South American studies. Studies were excluded if they assessed unrelated topics and herbs other than *Ilex paraguariensis* or were written in a language other than English. As a result of this targeted search, 77 records were obtained from PubMed and 65 results from the SciELO database. Duplicates were removed, and articles were screened by titles and abstracts. Thirty-three publications were selected and subjected to full-text analysis. Publications that were relevant in terms of a significant contribution to knowledge, as well as key findings and mechanistic explanations, were also added manually. Included publications were identified by screening the reference lists of eligible articles to capture relevant primary studies not retrieved by the database search. As a result, 42 publications were selected and used in this review. The search strategy and study selection have been depicted in Figure 1.

This review has several limitations. Overall, the available evidence is constrained by a predominance of small randomized controlled trials, the analysis of surrogate rather than hard clinical endpoints, and a substantial proportion of mechanistic data derived from preclinical models. This limitation was, however, partly mitigated by preferentially drawing on evidence from randomized controlled trials, clinical trials, and meta-analyses rather than from narrative reviews, which strengthens the reliability of the clinical conclusions drawn in this work. Additionally, the search was restricted to English-language records. Although research on *Ilex paraguariensis* is substantially rooted in South America, only several records meeting the eligibility criteria were identified in other languages (Spanish and Portuguese); given this small number, non-English records were not included, and we acknowledge that this restriction may have led to the omission of a limited amount of regionally published evidence. As a narrative rather than systematic review, this work was not intended to provide an exhaustive synthesis of all available literature but rather a critical and interpretive overview of the current evidence. Although the papers used in this review were selected by relevance, a PRISMA-informed approach was used to lower the risk of bias in this article. Despite this, we strongly emphasize the current evidence gaps.

Bioavailability and gut microbiota metabolism

One of the most important aspects of the biological activity of YM is the limited bioavailability of its bioactive compounds. Despite their high abundance, most native, unmetabolized phytochemicals are poorly absorbed in the stomach and small intestine, resulting in approximately two-thirds of its chlorogenic acids reaching the colon intact [15]. The colonic microbiota subsequently metabolizes these compounds, particularly into dihydrocaffeic and dihydroferulic acids, generating metabolites with greater bioavailability and bioactivity [15]. Interestingly, the interaction between YM and the gut microbiota is bidirectional. On the one hand, microbial metabolism enhances the bioavailability of biologically active compounds; on the other hand, YM-derived phytochemicals modulate the gut microbial composition by selectively promoting the growth of *Lactobacillus* and *Prevotella* spp., thereby supporting the prebiotic properties of YM [9]. It has been found that YM directly increases GLP-1 serum levels in vivo yet fails to increase GLP-1 secretion in L-cells, which are predominantly responsible for its production. These findings lead to a conclusion that YM constituents may be converted into bioactive metabolites that subsequently stimulate GLP-1 secretion from L-cells. This intermediate step may involve the colonic biotransformation described above: gut bacteria convert ferulic acid into dihydroferulic acid, a microbial metabolite that significantly increased GLP-1 secretion from L-cells, whereas whole YM extract had no such effect. Although microbial biotransformation plays a central role, it is not the sole mechanism involved, as chlorogenic acid can also stimulate GLP-1 secretion directly from L-cells, independently of microbial metabolism [16]. Additionally, it has been shown that YM usage increases GLP-1 levels without increasing GIP, which is particularly relevant because GIP exerts lipogenic effects, which could potentially counteract the beneficial metabolic effects of YM [16].

Glucose metabolism

The available literature suggests that YM may influence glucose metabolism. A meta-analysis of 13 randomized clinical trials (RCT) has shown a significant decrease in postprandial glucose levels (mean difference {MD}, -12.76 mg/dL; 95% CI, -16.78 to -8.74), glycated hemoglobin (HbA1c) (MD, -0.37%; 95% CI, -0.56 to -0.18), and the homeostatic model assessment (HOMA) index (MD, -0.24; 95% CI, -0.37 to -0.11) [14]. Interestingly, the meta-analysis found no significant effect of YM on fasting plasma glucose or fasting insulin levels, suggesting that its effect may be more relevant to postprandial and long-term glycemic control, as reflected by glycated hemoglobin (HbA1c), than to fasting glycemia [14]. In contrast, Bravo et al. found in their randomized controlled trial that moderate YM intake (9 g of leaves per day for eight weeks) significantly reduced fasting plasma glucose levels in both hypercholesterolemic and non-hypercholesterolemic individuals. Furthermore, improvements in insulin resistance and insulin sensitivity were also observed in participants receiving YM [8]. However, it should be emphasized that the meta-analysis by Li et al. [14] does not support the overall effect reported by Bravo et al. [8].

Moreover, an RCT by Gebara et al. also did not confirm YM’s effect on glycemia (fasting glucose and oral glucose tolerance test {OGTT}) in men with ≤1 risk factor of metabolic syndrome. However, a statistically significant decrease in fasting glucose (-0.57 ± 0.11 mmol/L; p < 0.0002) was observed among the mate group between days 0 and 28. This result does not confirm the effect of YM on fasting glucose, because within-group change did not reach significance relative to placebo [17].

Taken together, the available evidence suggests that the effects of YM on glucose metabolism range from negligible to more pronounced in individuals with impaired fasting glucose or dyslipidemia [8,14,17]. The effect on glycemia clearly requires more in-depth studies, due to discrepancies in available studies.

As discussed above, the effects of YM on glucose metabolism appear to be strongly influenced by an individual’s baseline glycemic status. This observation is supported by the meta-analysis conducted by Li et al., in which a significant effect was identified in the subgroup of participants with dyslipidemia. In contrast, no significant effect was observed in healthy, athletic, or normoglycemic cohorts, suggesting that YM may normalize dysregulated glucose homeostasis rather than reduce blood glucose below physiological levels [14].

It is also necessary to further delineate species-dependent differences in the effects of YM on incretin-related mechanisms. In mouse models, YM increased GLP-1 secretion without affecting GIP levels [16], whereas in humans, GLP-1 concentrations remained unchanged, while GIP and ghrelin levels were reduced [8]. Despite these discrepancies, the reduction in GIP, a lipogenic incretin, may be metabolically advantageous. These interspecies differences in physiological responses to bioactive compounds present in YM between preclinical animal studies and human trials represent an important evidence gap that warrants further investigation.

Multiple mechanisms have been proposed to explain the effects of YM on glycemic regulation. However, most of these studies have been conducted in murine or rat models, limiting the direct extrapolation of these findings to humans. Proposed mechanisms include the modulation of insulin signaling pathways and the regulation of hepatic gluconeogenesis [18]. In addition, phenolic compounds present in YM have been proposed, based on in vitro and animal studies, to inhibit α-amylase and α-glucosidase activity, interact with sodium-glucose cotransporter 1 (SGLT1), promote GLUT4 translocation, and enhance peripheral glucose uptake [19]. However, this mechanism would probably affect OGTT results in humans, and as Gebara et al. showed in their study, OGTT results were not affected by YM consumption [17].

Despite these promising findings, the available evidence should be interpreted with caution, as clinical data supporting the beneficial effects of YM in humans remain limited. Nevertheless, among the proposed cardiometabolic effects of YM, its influence on glucose metabolism appears to be supported by the most comprehensive mechanistic evidence.

Vascular function and blood pressure

The available literature suggests that *Ilex paraguariensis* exerts beneficial effects on vascular function and modulates inflammation and oxidative stress. Previous studies have distinguished between the acute and chronic effects of YM infusion consumption. Filipini et al. [20] demonstrated that a single dose of YM infusion, regardless of whether it was consumed hot or cold, did not significantly affect endothelial function, as assessed by flow-mediated dilation (FMD), which is a marker of vascular function associated with cardiovascular risk and mortality [21]. Furthermore, no significant changes were observed in heart rate or heart rate variability (HRV) in healthy individuals. The authors proposed that the absence of an acute vascular effect may be explained by the phytochemical composition of YM infusion, particularly its low catechin content, as catechins have well-established endothelial protective properties [10]. In contrast, the chronic consumption of YM infusion (5 g/day for six weeks) was associated with significant improvements in vascular function. In a randomized, double-blind clinical trial involving 142 participants with increased blood viscosity, Yu et al. demonstrated improved microcirculation, as assessed by nailfold capillaroscopy. Moreover, YM supplementation significantly reduced whole blood viscosity, plasma viscosity, and the erythrocyte sedimentation rate (ESR) coefficient. YM also favorably modulated the balance between platelet-derived vasoactive mediators by increasing prostacyclin levels (6-keto-PGF1α), a potent vasodilator and inhibitor of platelet aggregation, while reducing thromboxane B₂ (TXB₂), which exerts opposing prothrombotic and vasoconstrictive effects. This shift toward a higher prostacyclin-to-thromboxane ratio is considered beneficial for maintaining an antithrombotic vascular phenotype [22]. Since elevated blood viscosity is an established risk factor for both atherosclerosis and thrombosis, its reduction represents a mechanistically relevant cardiovascular benefit [23].

Hypertension is one of the most important cardiovascular risk factors [24]. The evidence of YM effect on blood pressure is scarce and remains unclear. Bravo et al. showed that YM consumption was associated with a significant decrease in systolic blood pressure (SBP) and diastolic blood pressure (DBP) (SBP, -8.2 mmHg; DBP, -6.63 mmHg) in the hypercholesterolemic group. Interestingly, YM also lowered BP in the healthy (normocholesterolemic) group, although the changes were more subtle (SBP, -2.84 mmHg; DBP, -0.9 mmHg); however, it must be emphasized that in both groups, the baseline blood pressure was normal [8]. Additionally, Filipini et al. showed that acute YM consumption does not affect blood pressure [20].

Inflammation and oxidative stress

Evidence also supports an anti-inflammatory effect of *Ilex paraguariensis*. Mechanistically, these effects are largely attributed to chlorogenic acid, which has been shown to inhibit the NF-κB and MAPK signaling pathways, as well as suppress NF-κB activation and reduce the production of adipose tissue-derived inflammatory cytokines [8,18]. Following YM consumption, high-sensitivity C-reactive protein (hs-CRP) concentrations decreased by approximately 22% and 40% in normocholesterolemic and hypercholesterolemic individuals, respectively [8], and by 50% in a group with at most one criterion of metabolic syndrome [17]. In addition, significant reductions were observed in circulating levels of tumor necrosis factor-α (TNF-α), interferon-γ (IFN-γ), monocyte chemoattractant protein-1 (MCP-1), macrophage inflammatory protein-1β (MIP-1β), and the colony-stimulating factors granulocyte (G)-CSF and granulocyte-macrophage (GM)-CSF, with decreases ranging from approximately 19.5% to 65.9% [8]. Also, interleukin-6 (IL-6) reduction by 19% was observed [17]. These findings are particularly relevant because MCP-1 and MIP-1β are abundantly expressed within atherosclerotic plaques, where they promote monocyte recruitment and sustain vascular inflammation [8,25,26]. Consequently, their reduction provides a mechanistic link between the anti-inflammatory properties of YM and the attenuation of atherogenesis [25,26]. It is noteworthy, however, that YM consumption did not significantly affect the circulating concentrations of the adhesion molecules vascular cell adhesion molecule-1 (VCAM-1) and intercellular adhesion molecule-1 (ICAM-1), nor did it alter interleukin-2 (IL-2) levels. These findings suggest that the anti-inflammatory effects of YM are selective rather than broadly suppressive [8].

YM has also been shown to influence oxidative stress and antioxidant capacity due to high contents of flavonoid and phenolic compounds [13]. Clinical studies have demonstrated that YM increases serum antioxidant capacity, as assessed by the ferric reducing ability of plasma (FRAP) and oxygen radical absorbance capacity (ORAC) assays, while reducing malondialdehyde (MDA) concentrations, a well-established biomarker of lipid peroxidation [8]. However, it should be noted that although the reduction in MDA reached statistical significance, the magnitude of this effect was modest and close to the threshold of significance. The antioxidant properties of YM have also been linked to its effects on paraoxonase-1 (PON-1), a high-density lipoprotein (HDL)-associated enzyme with potent antioxidant activity. In the study by Balsan et al., YM supplementation increased PON-1 activity by 9.7% (p = 0.005), and this increase was significantly associated with an elevation in HDL cholesterol concentrations (p = 0.004) [27]. This finding is mechanistically relevant because PON-1 protects low-density lipoprotein (LDL) particles against oxidative modification, thereby limiting one of the key processes involved in the initiation and progression of atherosclerosis [28]. Despite these promising findings, the antioxidant properties of YM should be interpreted with caution. Evidence from experimental murine models suggests that its overall antioxidant effect is relatively limited. Valença et al. described the antioxidant activity of YM as “rather limited” and reported no significant changes in superoxide dismutase (SOD) activity following YM administration [29].

Despite the potentially beneficial effects of yerba mate (YM) on vascular function, inflammation, oxidative stress, and hemostasis described above, evidence also suggests that YM may exert unfavorable effects on plasminogen activator inhibitor-1 (PAI-1), a major endogenous inhibitor of fibrinolysis. As reported by Bravo et al., YM supplementation was associated with increased PAI-1 concentrations in participants with hypercholesterolemia, a change that may theoretically promote a prothrombotic state [8]. It should be emphasized that the available studies have evaluated surrogate markers, which only allow the prediction of a potential beneficial effect on cardiovascular risk rather than the confirmation of such an effect. Data on cardiovascular endpoints remain unavailable.

Lipids, adiposity, and body weight

Hypercholesterolemia and other disorders of lipid metabolism are well-established and, importantly, modifiable risk factors for cardiovascular disease, such as atherosclerosis, stroke, and ischemic heart disease [30]. An elevated concentration of cholesterol carried in low-density lipoproteins (LDL-C), together with a reduced concentration of cholesterol associated with high-density lipoproteins (HDL-C), is regarded as an independent predictor of cardiovascular events (CVEs). Maintaining appropriate concentrations of these parameters constitutes an important goal in both the primary and secondary prevention of atherosclerosis. At present, statins are the most widely used class of cholesterol-lowering drugs. Both statins and agents with a different mechanism of action have their limitations, adverse effects that compromise treatment tolerance, or insufficient lipid-lowering efficacy that prevents the intended therapeutic target from being reached. This compels researchers to seek new treatment strategies, including those employing substances of plant origin [31,32].

Among all lipid parameters, the effect of YM is best documented for total cholesterol (TC), LDL-C, and triglycerides (TG). The magnitude of this effect depends on three main variables: the experimental model (in vivo rodent studies versus clinical studies in humans), the form and dose of the preparation, and the baseline status of the study population (normal cholesterol, abnormal cholesterol, or induced hyperlipidemia).

Total Cholesterol and LDL-C

Balzan et al. studied male rats with hyperlipidemia induced by a high-fat diet (HFD for 30 days). For 30 days, the animals received a standardized hydroethanolic extract of *Ilex paraguariensis* leaves (HEIP) and its n-butanol fraction (n-BFIP), which was enriched in chlorogenic acid derivatives; their content in the n-BFIP fraction was roughly three times higher than in a traditional infusion. Doses of 200-800 mg/kg/day lowered TC to a range of 70-104 mg/dL, though significance varied by dose. A clinically meaningful LDL-C reduction of 30-31 mg/dL was achieved only with n-BFIP at 200-400 mg/kg/day. The efficacy of this extract was comparable to that of reference simvastatin at 20 mg/kg/day [33]. Consistent results were also obtained in 40 male Swiss mice fed an HFD for 16 weeks and then given a YM infusion. Over eight weeks, TC decreased by a mean of 23% and LDL-C by 30% [34]. The most recent evidence for the lipid-lowering effect of YM comes from Tolouei et al. The study was conducted in male mice with obesity induced by a 16-week HFD. The animals then received a standardized aqueous extract of IP leaves for eight weeks. Liraglutide, a GLP-1 receptor agonist used to treat diabetes and obesity, served as the positive control. In the negative control group, which received water only, the HFD produced a significant increase in TC compared with animals fed standard chow. In HFD-fed animals, by contrast, the standardized IP leaf extract prevented this increase, with an effect comparable to that of liraglutide [3].

All the findings presented above come from animal models. Data from clinical studies in humans are also available. The lipid-lowering effect in humans is small but statistically significant. The broadest clinical evidence comes from de Morais et al. [32]. The investigators divided 102 participants (36 men and 66 women) into three groups: individuals with normal cholesterol, individuals with untreated hyperlipidemia, and individuals with hyperlipidemia treated with statins. All participants consumed 330 mL of YM infusion three times daily. The largest reduction in cholesterol occurred in participants who had hypercholesterolemia and were taking statins at study entry; in this group, LDL-C fell by 13.1% after 40 days of regular YM consumption. The authors noted that the response to YM varied considerably between individuals: in the statin-treated dyslipidemic group, LDL-C decreased by as much as 45% in some participants. The study also suggests a correlation between baseline cholesterol concentration and the magnitude of the reduction: the higher the baseline LDL-C, the stronger the cholesterol-lowering effect. In a randomized, double-blind study, Kim et al. administered a standardized aqueous extract of IP leaves in capsule form to 30 obese Korean participants for 12 weeks. Although no statistically significant changes were observed in any lipoprotein fraction, the observed changes were inconclusive. HDL-C trended favorably, but TC, LDL-C, and TG all increased. Despite their relatively small sample sizes, these studies support the further investigation of YM as a potential adjunct in the treatment of hypercholesterolemia [6].

Triglycerides (TG)

The effect of YM on triglyceride concentrations differs substantially between animal and human studies. In animal models, the TG-lowering effect is clearly apparent. In hyperlipidemic Syrian hamsters, a YM infusion lowered TG by 36% [30]. In Swiss mice fed an HFD, YM extract reduced TG to the level observed in animals fed a normal diet [34,35]. In human studies, by contrast, an effect of YM on TG has not been confirmed [6]. Thus, the TG-lowering effect that is well-documented in animals does not translate to the clinical setting. This discrepancy most likely reflects differences in the kinetics of triglyceride-rich lipoprotein metabolism between rodents and humans.

HDL-C

Unlike the consistent reduction in LDL-C, the effect of YM on HDL-C is not uniform and appears to depend on the baseline lipid profile and the experimental model. Findings from animal models have been inconsistent. The most pronounced effect on this lipoprotein fraction was described by Barroso et al. [36]. In their study in mice, HDL-C fell from 43.8 to 19.8 mg/dL in the group fed an HFD, whereas no such decrease occurred in the group that received an HFD together with a YM infusion. In other animal models, YM did not alter HDL-C levels and acted selectively on the atherogenic fractions. In the study by Gao et al., a YM infusion significantly lowered TC, LDL-C, and TG in hyperlipidemic hamsters, while HDL-C remained unchanged [30]. Martins et al. reported similar findings [34].

In human studies, the most favorable effects were observed in individuals with an abnormal lipid profile. In the study by de Morais et al., a YM infusion raised HDL-C by 4.4% in individuals with dyslipidemia and by 6.2% in patients treated with a statin [32]. Also, Gebara et al. described a 10% increase in HDL-C concentration in subjects with an intermediate to high Framingham risk score [17].

Body Weight

Evidence on the anti-obesity effect of YM in humans remains inconsistent. A meta-analysis of clinical trials reported significant reductions in body weight, body mass index (BMI), and waist circumference [37], whereas a more recent meta-analysis restricted to randomized controlled trials found no significant effect on BMI or waist circumference [14]. This discrepancy likely reflects differences in the included trials, populations, and dosing regimens. Preclinical data provide only partial support: in rats fed a high-fat diet, *Ilex paraguariensis* extract prevented body-weight gain and lowered plasma triglycerides, although it paradoxically increased the hepatic expression of lipogenic genes (*Fas* and *Scd1*) [35].

Hormonal Balance and Menopause

An additional and relatively recent line of research on YM concerns its influence on hormonal balance during the menopausal period. The decline in estrogen levels increases the risk of metabolic disturbances (atherosclerosis, dyslipidemia, and metabolic syndrome) and, consequently, cardiovascular risk [38,39]. Hormone replacement therapy not only can alleviate the consequences of menopause but also carries a risk of adverse effects, particularly breast cancer and thrombotic events [38]. This has prompted the search for plant-based substances that could favorably influence metabolism in this population [12].

In a study by Andrade et al. using a rat model (ovariectomized rats), eight weeks of YM administration reduced body-weight gain and brought triglyceride, total cholesterol, and LDL concentrations to levels close to those of the control group. YM also reduced adipocyte hypertrophy in the ovariectomized group, decreasing adipocyte size toward control values, while the reported increase in uncoupling protein 1 (UCP1) content suggests a browning of white adipose tissue (WAT) toward a brown-like (beige) phenotype [12]. The browning of WAT is beneficial for lipid and glucose metabolism, because beige adipocytes are metabolically more active than white adipocytes [40]. Importantly, YM appeared to mitigate the metabolic consequences of estrogen deficiency rather than to act through an estrogenic mechanism.

Another line of preclinical evidence comes from the study by Rocha et al., which also supports the finding that YM reduces body-weight gain in ovariectomized rats. An additional reported effect was the restoration of glucose oxidation and lipid synthesis in visceral (and retroperitoneal) adipose tissue. Notably, the effect of YM on adipose tissue may be sex-specific, modulating lipogenic pathways in women and lipolytic pathways in men [41].

Given the preclinical nature of studies, the evidence for a beneficial effect of YM on postmenopausal metabolic status remains theoretical and cannot be directly extrapolated to humans.

Safety and risk considerations

Despite several studies reporting positive effects of yerba mate extracts on metabolism and the cardiovascular system, there are still potential risks associated with its consumption. The infusion contains pharmacologically active substances (methylxanthines, tannins, and numerous phenolic compounds) that, when consumed regularly in high doses, could potentially alter hepatic metabolism or potentiate the effects of other stimulants [5]. The data presented below summarize the available evidence on the tolerability of standardized yerba mate extract in preclinical studies, as well as the potential risks associated with its regular consumption.

Hepatic Effects

In diabetic rats treated with yerba mate extract (DM group), there was a significant increase in ALT compared to the other groups and an increase in AST compared to the control group. The authors suggest the following conclusion: the administration of yerba mate extract to diabetic individuals may place additional strain on the liver [5].

Systemic Toxicity and Genotoxicity

A complete safety testing program for the standardized extract TI-076 was conducted in accordance with the Organisation for Economic Co-operation and Development and Good Laboratory Practice (OECD/GLP) guidelines. The following results were obtained: the Ames test (a bacterial reverse mutation assay that uses Salmonella typhimurium strains to detect whether a substance induces genetic mutations and is therefore used as a standard screening tool for mutagenic and potential carcinogenic risk) and the micronucleus test were negative. The maximum tolerated dose of the standardized extract was established at up to 5000 mg/kg, with no deaths or significant changes. Additionally, biochemical deviations were recorded as reversible, and no cardiovascular disturbances were observed after the administration of 2500 mg/kg [3].

*Central Nervous System* (*CNS)/Stimulant Effects*

The extract contains substantial amounts of theobromine, theophylline, and caffeine, in a well-absorbed form without accumulation, which indicates that these compounds reach systemic circulation efficiently after oral intake and do not build up in the body with repeated dosing. This supports the thesis of a favorable pharmacokinetic profile of YM that reduces the risk of long-term toxicity or unpredictable drug levels associated with chronic use. TI-076 was shown not to significantly affect the pharmacokinetics of midazolam [3]. However, in the Irwin test (a standardized behavioral screening assay used to detect CNS effects of a substance), it increased arousal, muscle tone, and respiratory rate at all tested doses [3]. Caution should be exercised when combining caffeine with other stimulants, since these behavioral findings indicate that the extract’s methylxanthines exert a genuine stimulant effect on the central nervous system; when combined with additional stimulant substances, such effects could accumulate and increase the risk of excessive CNS or cardiovascular activation [3].

Risks Related to Preparation Method

An additional risk factor is the method of preparing the infusion itself, specifically the high temperature; this increases the formation of carcinogenic compounds (polycyclic aromatic hydrocarbons {PAHs}), and the beverage’s high temperature itself may cause damage to the gastrointestinal mucosa [42].

In summary, the available preclinical evidence suggests that yerba mate is generally well-tolerated and associated with a relatively low risk of adverse effects. The greatest concern appears to involve liver function in individuals with diabetes who consume yerba mate regularly, although the available evidence on this issue remains inconsistent [3,5]. It should be emphasized that these findings are derived from animal studies; therefore, caution is warranted when extrapolating them to humans. As discussed previously, results obtained in animal models do not always correspond to those observed in clinical studies involving human subjects [8,19].

Evidence gaps and research priorities

The most important evidence gap that can be identified on the basis of this literature review is the complete absence of data on hard cardiovascular endpoints for YM consumption [14,42]. To date, only surrogate markers have been studied, which do not allow an unequivocal assessment of the effect of YM on cardiovascular risk [8,22,33]. In addition, it must be emphasized that human studies are scarce and often conducted in groups of healthy subjects, which clearly limits the transferability of the results to individuals with metabolic disorders. It is also worth noting that the evidence for an effect on glycemia remains insufficient, given that the data subjected to analysis were not extensive [14]. A further aspect that hinders comparison between studies is the lack of standardization of YM extracts. Despite attempts to address this, the results obtained with a standardized extract unfortunately cannot be straightforwardly extrapolated to an ordinary YM infusion [3]. Exposure time appears to be a key factor responsible for the effect of YM; however, a direct comparison of acute and chronic use within a single study devoted exclusively to this question is lacking [20,22]. The confirmation of the role of the gut microbiota in the metabolism of the substances contained in YM within the human intestine is also lacking. Studies conducted to date have been carried out exclusively in rodents [9,16]. Moreover, differences in the incretin response between species (humans versus rodents) are apparent, which constitutes an additional argument undermining the extrapolation of results obtained in rodents to humans. A recognizable evidence gap is also the safety profile of YM, which requires confirmation in a human model.

## Conclusions

Considering all the effects of YM, the strongest and best documented is its impact on carbohydrate metabolism. This effect is mechanistically substantiated and comprises a reduction in postprandial glycemia, HbA1c, and the HOMA index. The effect on glycemia appears to be supported by coherent mechanisms of insulin and incretin signaling. Based on weaker evidence is the antioxidant action, which translates into a reduction in inflammatory parameters and, indirectly, into an increase in HDL concentration, potentially exerting an anti-atherogenic effect. A beneficial effect of YM on the rheological properties of blood and on microcirculation has also been postulated, although current knowledge in this respect is exceedingly scarce. Evidence for a beneficial effect of YM on blood pressure, lipid profile, and body weight remains inconsistent. YM seems to have beneficial effects on postmenopausal metabolic status, although the data are derived from rat models and cannot be extrapolated to humans. The current evidence allows the cardiometabolic action of YM to be regarded as biochemically and mechanistically plausible yet still clinically unconfirmed, which warrants further, more in-depth research.
